# A multi-stage feature selection method to improve classification of potential super-agers and cognitive decliners using structural brain MRI data—a UK biobank study

**DOI:** 10.1007/s11357-024-01458-9

**Published:** 2024-12-10

**Authors:** Parvin Mohammadiarvejeh, Mohammad Fili, Alice Dawson, Brandon S. Klinedinst, Qian Wang, Shannin Moody, Neil Barnett, Amy Pollpeter, Brittany Larsen, Tianqi Li, Sara A. Willette, Jonathan P. Mochel, Karin Allenspach, Guiping Hu, Auriel A. Willette

**Affiliations:** 1 University of Maryland Medical System, Linthicum, MD USA; 2 Lighthouse Institute, Chestnut health Systems, Chicago, IL USA; 3 Department of Medicine, University of Washington, Seattle, WA USA; 4 Department of Food Science and Human Nutrition, Iowa State University, Ames, IA USA; 5 Department of Human Development and Family Studies, Iowa State University, Ames, IA USA; 6 Bioinformatics and Computational Biology Graduate Program, Iowa State University, Ames, IA USA; 7 Department of Behavioral Science, Division of Cancer Prevention and Population Sciences, University of Texas MD Anderson Cancer Center, Houston, TX USA; 8 Genetics and Genomics Graduate Program, Iowa State University, Ames, IA USA; 9 IAC Tracker Inc., Ames, IA USA; 10 Department of Biological Sciences, Iowa State University, Ames, IA USA; 11 Department of Pathology, College Veterinary Medicine, University of Georgia, Athens, GA USA; 12 Robert Wood Johnson Medical School, Rutgers University, Clinical Academic Building, 125 Paterson St., New Brunswick, NJ 08901 USA; 13 School of Industrial Engineering and Management, Oklahoma State University, Stillwater, OK USA

**Keywords:** Aging, Cognitive decline, Machine learning, FreeSurfer, Structural MRI

## Abstract

Cognitive aging is described as the age-related decline in areas such as memory, executive function, reasoning, and processing speed. Super-Agers, adults over 80 years old, have cognitive function performance comparable to middle-aged adults. To improve cognitive reserve and potentially decrease Alzheimer’s disease (AD) risk, it is essential to contrast changes in regional brain volumes between “Positive-Agers” who have superior cognitive performance compared to their age peers but are not 80 years old yet and aging adults who show cognitive decline (i.e., “Cognitive Decliners”). Using longitudinal cognitive tests over 7–9 years in UK Biobank, principal component analysis (PCA) was first applied to four cognitive domains to create a general cognition (GC) composite score. The GC score was then used to identify latent cognitive groups. Given cognitive groups as the target variable and structural magnetic resonance imaging (sMRI) data and demographics as predictors, we developed a multi-stage feature selection algorithm to identify the most important features. We then trained a Random Forest (RF) classifier on the final set of 54 selected sMRI and covariate predictors to distinguish between Positive-Agers and Cognitive Decliners. The RF model achieved an AUC of 73%. The top 6 features were age, education, brain total surface area, the area of pars orbitalis, mean intensity of the thalamus, and superior frontal gyrus surface area. Prediction of cognitive trajectory types using sMRI may improve our understanding of successful cognitive aging.

**Supplementary Information:**

The online version contains supplementary material available at 10.1007/s11357-024-01458-9.

## Introduction

Cognitive aging is the natural decline in various cognitive domains as people get older [1]. There is a broad literature on age-related decline in multiple cognitive functions such as processing speed, working memory, long-term memory, and executive function [2–7]. Although significant controversy exists about the age at which cognitive decline begins, general processing ability declines from early adulthood [8]. Additionally, there are substantial decreases in episodic memory and executive function long before the diagnosis of Alzheimer’s disease (AD). The decline in both cognition forms is effective at recognizing early AD risk [9, 10]. Nevertheless, some late-life adults show resistance to age-related decline in episodic memory. Super-Agers, adults over 80 years old, have cognitive function performance comparable to middle-aged adults (50 to 60 years old), particularly in memory [11]. Similarly, a group of mid- to late-life adults in the UK Biobank showed a cognitive gain over time in Fluid Intelligence (FI) scores related to verbal and numerical reasoning [12]. Cognitive decline is an essential element of public health that influences independent living and successful aging [13, 14]. Therefore, investigating the neurobiological differences between adults with cognitive gains and natural cognitive decline may reveal the underlying biomarkers of superior performance.

Structural magnetic resonance imaging (sMRI) measures the area, thickness, volume, and integrity of brain areas. sMRI is often used to better diagnose participants along the AD continuum [15]. Moreover, sMRI data is widely used to recognize the most resilient brain areas in Super-Agers and adults with cognitive reserve [15]. In this matter, Super-Agers showed significantly thicker cerebral cortex than age-matched adults, but no significant difference with individuals aged 50 to 65 years old. Moreover, Super-Agers had significantly thicker left anterior cingulate cortex than both control groups [16], as well as higher grey matter volume in bilateral anterior thalamus, bilateral hippocampus, the amygdala, entorhinal cortex, parahippocampal gyrus, and fusiform gyrus [17]. Further, for annual percentage change, Super-Agers versus elder controls had significantly less whole cortex volume loss [18]. Aging adults with no loss in memory and executive function had thicker cerebral cortexes and preserved hippocampal volume [19]. Finally, Super-Agers demonstrated greater volume in the hippocampus, putamen, thalamus, and pallidum compared to adults with natural cognitive aging [20].

Recently, machine learning techniques have been used to discriminate Super-Agers and cognitive decliner adults. One study used longitudinal memory and a few non-memory tests to label the adults as either Super-Agers or typical older adults in a small sample size, then used Random Forest (RF) to classify two cognitive groups using 89 predictors including demographic, lifestyle, blood, and brain biomarkers [21]. This study reported a slower rate of grey matter atrophy in Super-Agers than in natural cognitive decliner adults. This research suggested adding more related predictors in future studies as it obtained 66.4% accuracy in the classification [21].

As mentioned above, most studies have used various cognitive tests individually or simultaneously to identify Super-Agers in a relatively small sample size and compare some key brain areas between Super-Agers and normal cognitive decliners. In other words, previous studies applied the typical thresholds to the different cognitive exams to label the Super-Agers. Then, they statistically compared a few brain areas between the Super-Agers and normal cognitive decliners. Yet, no large-scale study has presented a combinative structure of multiple cognitive domain outcomes concerned with brain structure segmentation to identify Super-Agers and adults with declining cognition over time. The current longitudinal study in the UK Biobank used principal component analysis (PCA) to combine various cognitive tests to classify different cognitive trajectory types based on visual declarative memory, processing speed, prospective memory, verbal and numeric reasoning, and executive function [22].

In this study, we utilized T1 sMRI features and demographic data to distinguish between adults exhibiting superior cognitive performance, referred to as “Positive-Agers,” and those experiencing cognitive decline, referred to as “Cognitive Decliners.” The primary distinction between Positive-Agers and Super-Agers lies in age: Super-Agers are above 80 years of age, while Positive-Agers are younger. In our study, Positive-Agers range from 55 to 70 years at the baseline visit. Due to the high dimensionality of the feature space, we proposed a multi-stage feature selection algorithm to eliminate redundant features and identify the most resilient brain areas associated with successful cognitive aging. Subsequently, we extracted the influential predictors and analyzed their contributions to the classification outcomes.

The rest of the paper is organized as follows: the “Methods” section includes a detailed description of data collection and applied methods in cognitive trajectory labeling, feature selection, and classification. The “Results” section provides the results of statistical analysis and classification models. Finally, the “Discussion” section states the discussion, limitations, and future directions.

## Methods

In this study, participants were a part of the UK Biobank study. UK Biobank is one of the largest longitudinal biomedical databases in the world investigating the health of over half a million middle-aged to older UK adults [22]. Participants in our study were aged 55 to 70 at the baseline visit in 2006–2010 (i.e., Visit I). The first follow-up visit (i.e., second visit) was held in 2012–2013 (i.e., Visit II). Visits I and II included (1) informed consent; (2) touchscreen questionnaire; (3) verbal interview; (4) eye measures (i.e., visual acuity, refractometry, retinal optical coherence tomography, and eye surgery history); (5) anthropometric measures; and 6) blood/urine sample collection. Socio-demographic characteristics, occupation, lifestyle, and cognitive function were gathered by questionnaires using touchscreens or laptops. Two subsequent follow-up visits, i.e., the third visit (denoted as visit III) and the fourth visit (denoted as visit IV), were held starting in 2014 and 2019, respectively. Visits III and IV included the above five items and additional imaging for the brain, heart, and body [23]. In this study, we used an initial cohort of 8528 participants based on the available cognitive tests, sMRI, and demographic features.

### Demographic data

The demographics data includes information on age, sex, socioeconomic class, education level, and tobacco use. Age was defined as the age in years of each participant at their baseline visit. The socioeconomic class was defined by the participant’s average total household income. There were five options provided based on UK Biobank Data-Coding to be selected by participants in British pounds, which included “Less than 18,000,” “18,000 to 30,999,” “31,000 to 51,999,” “52,000 to 100,000,” and “Greater than 100,000.” These groups were labeled as “under,” “lower,” “middle,” “upper-middle,” and “upper class.” For education level, a categorical variable was used with the following levels: college or other higher-level status; post-secondary or vocational; secondary; and none of the previous ones. Finally, tobacco smoking status was defined as a categorical variable with levels: “never smoked,” “previously smoked,” and “currently smoking.”

### Cognitive function tests

Cognitive function tests in the UK Biobank study were operated as part of the fully automated touch screen questionnaire [22]. The cognitive function testing section of the UK Biobank data describes the administration and basic statistics [24]. Here, we focused on Reaction Time (RT), Fluid Intelligence (FI), Prospective Memory (PM), and Pair Matching Memory (PMM), which are associated with processing speed, verbal and numerical reasoning, prospective memory, and visual declarative memory, respectively [25]. It is worth noting that RT and PMM values were log(x)- and log(x + 1)-transformed, respectively.

### General cognitive ability

General Cognition (GC) can be derived from the cognitive function assessment scores while retaining most of the variability in the original exams’ scores [25–27]. One way to create a GC composite score is by using PCA [28]. A composite score that is derived by correlation coefficients in the first principal component accounts for the most variance in the cognitive tests and is considered the GC score [25].

In this study, we considered a longitudinal combination of the cognitive tests described in the “Cognitive function tests” section at visits I and III as the input for the PCA. Then we extracted the first component which accounted for 28% of the variance in the eight cognitive tests (see Supplementary Text A). This method is based on valid and comprehensive cognitive exams that are susceptible to age-related decline [25]. The composite scores extracted from the first principal component were considered as the GC score. Looking at the loading scores in Supplementary Table A.1, we can observe that regardless of the time points, weights corresponding to FI and PM tests are negative and the signs for PMM and RT exams are positive. This means that the higher the GC score, the lower the cognitive performance of an individual is, and vice versa. To make it more interpretable and easier to understand, we multiplied the GC score by − 1, so that larger GC scores are associated with a higher likelihood of being a Positive-Ager.

The first and third quartiles of the composite scores were then used as the thresholds to identify “Cognitive Decliner” and “Positive-Ager” classes. The remaining 50% of middle participants were considered “Cognitive Maintainers” (i.e., who showed relatively stable GC scores) and were dropped from the analysis. This enabled us to focus on extreme cognitive performance and identify the most resilient and non-resilient groups to age-related changes in brain areas. Additionally, this helps us compare positive agers against decliners and consequently find the brain regions associated with cognitive reserve.

### sMRI data

UK Biobank conducted structural imaging at visits III and IV. Imaging assessments were done at three centers on the same Siemens Skyra scanners with a standard Siemens 32-channel head coil [29]. UK Biobank imaging includes six modalities, including T1, T2-weighted, susceptibility-weighted MRI, resting functional MRI (rsfMRI), task functional MRI (tfMRI), and diffusion MRI (dMRI). Among these six modalities, T1, T2-weighted, and susceptibility-weighted MRI present anatomical and neuropathological structures of the brain. In this study, we used T1 at visit III, which is informative about the structure of the brain, depiction of the main tissue type, main anatomical landmarks, and tissue loss [30, 31]. From the T1 sMRI features, we used Freesurfer ASEG, Freesurfer desikan gw, Freesurfer desikan pial, Freesurfer desikan white, and Freesurfer desikan sub-segmentation (558 sMRI features in total).

### A multi-stage feature selection method

Data mining and machine learning algorithms have difficulty in dealing with high-dimensional data. This gets even worse when the feature space has noisy, irrelevant, or highly correlated features. While consuming a lot of computational resources, such cases often end up with poor model performances. One solution to such problems is to employ dimensionality reduction techniques, such as utilizing feature selection methods to improve the overall performance. This also helps to decrease memory usage and may reduce the overall run time [32, 33]. In this study, we had high-dimensional and highly correlated feature sets. Therefore, incorporating feature selection was necessary to have reliable and accurate predictive models.

We faced the following challenges during the analysis of sMRI data: (1) high correlation between features; (2) the high number of features even after correlation-based filtering; (3) very long training and tuning times; (4) discordance of the final feature subset among different traditional feature selection approaches such as heuristic algorithms, filter, wrapper, and embedded methods.

To address the above issues and have a reliable and accurate model, we designed a multi-stage feature selection algorithm. The proposed algorithm removed irrelevant features, addressed the multicollinearity problem in the data, decreased the overall run time, and improved the classification performance. The three stages of the proposed algorithm are summarized below:

#### Stage A

Let $\left\{\left({X}_{i}, {y}_{i}\right): {X}_{i} \in {R}^{p}, {y}_{i} \in \left\{0, 1\right\}, i=1, \dots , n\right\}$ be the training set, which consists of $n$ pairs of feature vectors of size $p$ and target values (binary, here). First, the algorithm drops the features for which the mutual information (MI) value, with respect to the response variable, is less than a specified threshold α. In the next step, the algorithm uses MI scores to sort the features in an ascending order and then calculates the correlation between the first feature and the second. If the pairwise correlation exceeds a certain threshold $\upbeta$, then the feature with the lower MI score will be dropped. Otherwise, the search will continue for the first feature until all remaining variables are explored. Then, the process is repeated on the next feature. This process continues until no feature with a high degree of correlation (i.e., Pearson correlation coefficient higher than the $\beta$) remains in the feature set.

#### Stage B

Using the L1-regularized Logistic Regression (L1-regularized LR), the algorithm computes the cross-validated area under the curve (AUC) for a range of regularization parameter values, $C \in (0, {C}_{M}]$, on the training set obtained from Stage A, in which ${C}_{M}$ is a large positive float. In this notation, $C$ is the inverse of regularization strength (often denoted as $\lambda$). In the next step, the smallest ${C}^{*}$ is selected for which no significant improvement in the cross-validated AUC in the interval $({C}^{*}, {C}_{M}]$ is recorded. Then, the algorithm picks the subset of features resulting from training the L1-regularized LR with regularization parameter ${C}^{*}$, on the training set.

#### Stage C

The algorithm employs the sequential feature selection (SFS) method to find the final set of best features. Bayesian optimization is used to ensure the optimal performance of the algorithm.

A detailed description of the proposed algorithm is provided in Supplementary Text B.

### Classification

Classification methods are supervised machine learning techniques that are designed to find a relationship between some class labels and the corresponding input features. The classification process includes two main steps: training and testing. In the training phase, a classifier model is trained on a proportion of the observations. The training phase may include the feature selection procedure and hyperparameters tuning. In the testing phase, the performance of the fitted model is evaluated using the rest of the data, which has not been seen by the model during the training [34].

In this study, we reported Random Forest (RF) and Support Vector Machine (SVM) classifiers to discriminate between the Positive-Ager and Cognitive Decline groups. RF is a powerful ensemble method consisting of many decision tree classifiers whose predictions are then aggregated into a final predicted class label using the majority vote rule. RF is robust against overfitting and multicollinearity. It provides feature importance ranking based on the mean decrease in the impurity, which shows the contribution of the features toward classification [35]. The second model used in this study is SVM which is a widely used classification technique founded on statistical learning theory to locate the decision boundaries of class labels. SVM uses linear or nonlinear decision surfaces based on the kernel function to separate the classes [34].

As the multi-stage feature selection algorithm incorporates L1-regularized LR and SFS, we compared the proposed algorithm against L1-regularized and SFS methods individually. For this purpose, we evaluated three feature selection algorithms (i.e., the multi-stage feature selection, L1-regularized LR, and SFS) combined with one of the RF or SVM classifiers.

## Results

### Demographic and data summaries

The final pool of participants after GC scoring and labeling consists of 3064 individuals who were labeled as either “Positive-Ager” or “Cognitive Decliner.” Table 1 shows the summary of statistics for age, demographic, and cognitive function tests for these participants.

**Table 1:** Summary of statistics for identified positive-agers and cognitive decliners (*N* = 3064)

Variable	Unit/range	Positive-ager *n* = 1684 (55%)	Cognitive decline *n* = 1380 (45%)	$P$-value^a^
Age I	Years	60 ± 3.5^**b**^	61.7 ± 3.9	** < 0.001**
Sex	No. (%)			
Female		686 (40.7) ^**c**^	736 (53.3)	** < 0.001**
Male		998 (59.3)	644 (46.7)	
Education level	No. (%)			
College/other higher level		1358 (80.6)	747 (54.1)	** < 0.001**
Post-secondary/vocational		220 (13.1)	246 (17.8)	
Secondary		95 (5.6)	208 (15.1)	
Other		11 (0.7)	179 (13)	
Social class	No. (%)			
Under		154 (9)	284 (20.6)	** < 0.001**
Lower		386 (23)	436 (31.6)	
Middle		522 (31)	370 (26.8)	
Upper-middle		488 (29)	238 (17.2)	
Upper		134 (8)	52 (3.8)	
Tobacco use	No. (%)			
Non-smoker		1015 (60.3)	824 (59.7)	0.86
Prior smoker		602 (35.7)	496 (36)	
Smoker		67 (4)	60 (4.3)	
FI I^d^	Total FIT score	8.7 ± 1.6	5 ± 1.5	** < 0.001**
FI III	Total FIT score	8.7 ± 1.6	4.9 ± 1.5	** < 0.001**
PM I	Integer {0, 1}	0.98 ± 0.1	0.64 ± 0.5	** < 0.001**
PM III	Integer {0, 1}	0.98 ± 0.1	0.60 ± 0.5	** < 0.001**
PMM I	Integer	1.13 ± 0.6	1.69 ± 0.6	** < 0.001**
PMM III	Integer	1.11 ± 0.6	1.70 ± 0.6	** < 0.001**
RT I	Milliseconds	6.22 ± 0.1	6.43 ± 0.2	** < 0.001**
RT III	Milliseconds	6.30 ± 0.1	6.52 ± 0.2	** < 0.001**

^a^*P*-values are obtained from NOVA for the continuous variables and chi-square test for the categorical variables. Values in bold font show statistical significance

^b^Mean ± SD

^c^Values in parentheses are percentages of a sub-group in each cognitive group

^d^FI scores are the number of questions answered correctly out of 13 questions, and the original scores were entered into PCA. PM is measured with binary integers; zero means wrong answer, and one means correct answer and original values were entered into PCA. PMM is measured by integers, which have a wide range; the log(x + 1)-transformed was used for the PCA, and the table shows the transformed statistics accordingly. RT is measured in milliseconds, which has a wide range; the log(x)-transformed was used for PCA, and the table shows the transformed statistics accordingly

### Model performance and feature importance

Table 2 compares the performance of different choices of classifiers (RF vs. SVM) along with feature selection algorithms (L1-regularized LR + SFS, SFS, and L1-regularized LR) on an independent test set (20% of data). In models with the “L1-regularized LR” feature selector, Bayesian optimization was used to tune the L1 regularization parameter and the classifier’s hyperparameters. In the feature selection mechanisms with “SFS” as part of it, optimization was used to tune the number of selected features by SFS and the classifier’s hyperparameters.

**Table 2:** Classification models performance

Classifier	Feature selection	${N}_{f}$	Time (s)	Accuracy	Precision	Recall	AUC
RF	L1-regularized LR	226	22	66	67	76	71
	SFS	64	65,028	66	67	77	72
	L1-regularized LR + SFS	54	47,591	67	68	79	73
SVM	L1-regularized LR	311	12	64	67	70	66
	SFS	84	63,680	64	66	73	70
	L1-regularized LR + SFS	40	44,813	67	69	77	71

${N}_{f}$ represents the number of features in the final subset selected by the specified feature selection model. It is worth noting that the filtering process in Stage A is used in the baseline models, too. Time shows the time per trial of hyperparameters tunning by Bayesian optimization in seconds. All values of accuracy, precision, recall, and AUC are in percentage

The proposed multi-stage feature selection algorithm, using either RF or SVM, showed better classification performance than baseline models. The proposed multi-stage feature selection with RF showed a 1–3% increase in accuracy, precision, recall, and AUC in comparison to the “L1-regularized LR” and “SFS.” In terms of the number of selected features, the proposed “L1-regularized LR + SFS” selected 54 features out of 354 (i.e., 354 is the number of remaining features after stage A), while “L1-regularized LR” selected 226 features. In terms of runtime, the proposed “L1-regularized LR + SFS” showed significantly less running time than “SFS.” The proposed multi-stage feature selection with SVM showed a 1–7% increase in accuracy, precision, recall, and AUC in comparison to the “L1-regularized LR” and “SFS.” In terms of the number of selected features, the proposed “L1-regularized LR + SFS” selected 40 features out of 354, while “L1-regularized LR” selected 311 features. In terms of tuning time per iteration, the proposed “L1-regularized LR + SFS” showed significantly less running time than “SFS.” In summary, the proposed “L1-regularized LR + SFS” feature selection model could select the most relevant features, improve the classification performance, and decrease the hyperparameter tuning process, which is time-consuming when working with large and high-dimensional datasets.

**Fig. 1:**
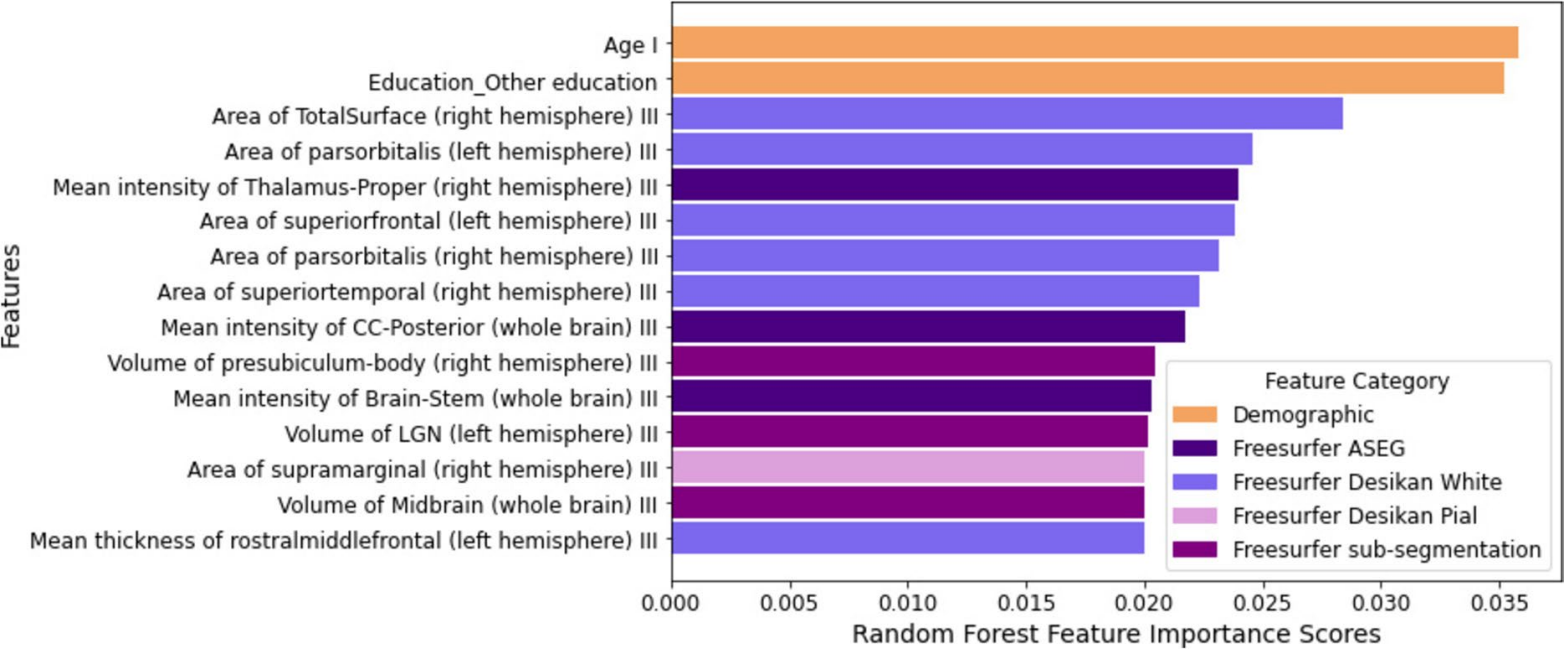
Feature importance ranking for the top fifteen features of the L1-regularized LR + SFS feature selection along with RF classifier. Colors represent feature categories (i.e., demographic and FreeSurfer types). For a given feature, the “I” and “III” symbols represent cohort visits: 2006–2010 (i.e., visit I) or 2014 (i.e., visit III)

In Fig. 1, the top 15 features were ranked according to feature importance obtained from the multi-stage feature selection algorithm with RF. A full feature importance ranking of all combinations has been provided in Supplementary Fig. 1.

Age and education were the top features selected. This is sensible, as age and education are, respectively strong risk or protective factors for AD [8, 36, 37]. For FreeSurfer features, regions included areas that show atrophy in AD, such as the superior and middle frontal gyrus, superior temporal gyrus, posterior cingulate cortex, and presubiculum. Surprisingly, several atypical regions are loaded that usually do not show atrophy or GM loss in MCI or early AD. These areas included the brainstem, the LGN of the thalamus corresponding to sight, and the midbrain.

**Fig. 2:**
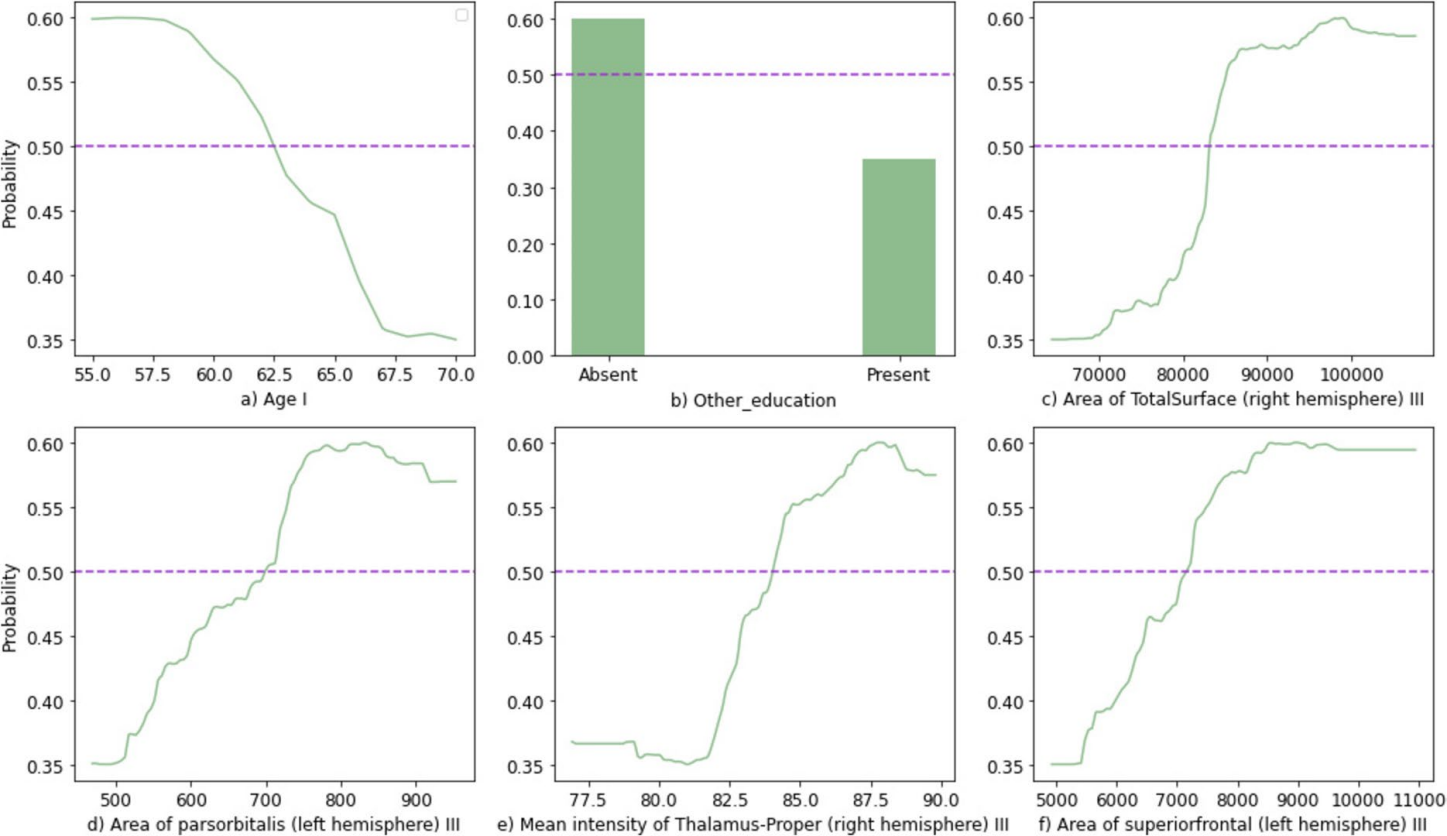
The partial dependence plots illustrate the relationship between levels of the given feature and the probability of being either in the Cognitive Decline group [0, 0.5), or the Positive-Ager group [0.5, 1.0]. The purple dashed line shows the 0.50 threshold separating Positive-Agers and Cognitive Decliners

It is important to know how levels of features correspond to the probability of being either Positive-Agers or Cognitive Decliners. Figure 2 shows the partial dependence plots of the top 6 features.

### Analysis of cognitive trajectories

**Fig. 3:**
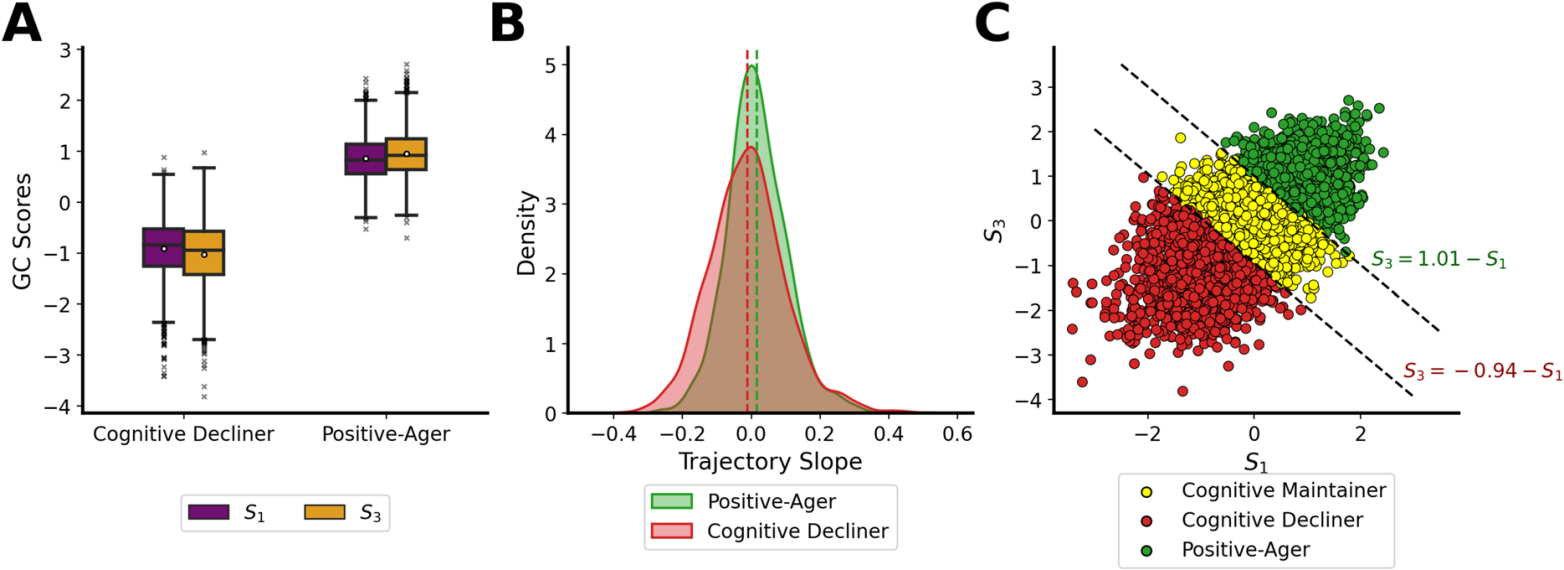
Analysis of GC scores and the corresponding trajectory slope. Subplot **A** Box plot of GS score at visits I and III for Positive-Agers and Cognitive Decliners. Subplot **B** Estimated kernel density plot of cognitive trajectory slope for Cognitive Decliners Vs. Positive-Agers. The green and red dashed lines are the mean trajectory slope for the Positive-Ager and Cognitive Decliner groups, respectively. Subplot **C** Scatter plot of GC score at the third visit against the GC score at baseline. Black dashed lines show the boundaries separating cognitive classes

While identifying extreme cognitive groups is insightful, analysis of cognitive trajectories may provide additional insights. For this reason, we calculated the GC score at visits I and III separately (denoted as ${S}_{1}$ and ${S}_{3}$) and computed the corresponding trajectory slope as the ratio of the difference in GC score (${S}_{3}-{S}_{1}$) to the time duration between these two visits (${t}_{3}-{t}_{1}$) for each participant. We then compared Positive-Agers against Cognitive Decliners with respect to GC scores at each visit and trajectory slopes (see Fig. 3). We observed several interesting findings: (1) there is a positive correlation between the GC scores at visits I and III; (2) there is a significant difference between the GC scores of Positive-Agers and Cognitive Decliners for both visits; (3) cognitive decliners are more likely to have negative trajectory slopes; and (4) average trajectory slope for Positive-Agers and Cognitive Decliners are 0.016 and − 0.010, respectively.

## Discussion

Although aging is generally accompanied by a decline in many cognitive processes, there is variation in timing and affected cognitive tasks. Some adults experience such declines from their early 20s onward, while other individuals have intact cognition regardless of age. Moreover, Positive-Agers who demonstrate cognitive abilities comparable to middle-aged adults support the idea of successful cognitive aging. In particular, it may be possible to enhance successful aging and prevent AD by better understanding how sMRI predictors explain changes between potential Super-Agers and Cognitive Decliners.

In this matter, we recently found that predominantly vascular and bioenergetic factors distinguished these latent groups with a high degree of accuracy over 7–10 years [12]. In the current study, we used PCA to combine multiple cognitive exam results and quantify GC. We then specified Positive-Ager and Cognitive Decliner groups using the GC scores obtained. In the next step, an RF classification model was trained on sMRI features and demographic data to predict the cognitive classes. To reduce the feature dimensionality and identify the most important predictors, we designed and applied a multi-stage feature selection algorithm. With the GC labels aggregating processing speed, verbal and numeric reasoning, prospective memory, and visual declarative memory, we achieved an AUC of 73% with 54 selected sMRI features. In the following, we discuss the top features identified in this study.

Age at baseline was the most important feature in classification. In general, age has a negative association with cognitive functions and is the most important risk factor in AD [38]. The second most important feature was years of education described as “Other,” which was used in the form of a binary variable. Previous studies found that higher levels of education lead to better cognitive performance in elders [36, 37, 39]. Similarly, according to Fig. 2, we found that people with other types of education, including college or other higher-level status, post-secondary or vocational, and secondary, were more likely to be Positive-Agers. The area of the total surface (right hemisphere) was the third most important predictor. The cerebral cortex is the outer layer of the brain covering the cerebrum and is formed predominantly by grey matter. Increases in cortical surface area, formed by cortical folding (called “gyrification”), enhance connectivity between regions. Gyrification pattern is governed by genetic factors but not age. There is a positive association between cognitive abilities and total surface area because of more processing capability and better connectivity provided by more neurons for ensemble processing. Therefore, cortical surface area is an important predictor of cognitive features [40–42]. Previous studies showed changes in the cortical surface area in lifespan and decreased surface area of different parts due to aging [43, 44]. Similarly, we found that a higher surface area in general corresponded to a higher probability of being a Positive-Ager.

The fourth most important feature was the area of pars orbitalis (left hemisphere). Pars orbitalis, pars triangularis, and pars opercularis are three parts constituting the inferior frontal gyrus (IFG). IFG is associated with important cognitive areas including executive function, emotional and social tasks, inhibition, and language processing [45, 46]. Pars orbitalis has a key role in language processing [47]. The cortical thickness of both left and right pars orbitalis is correlated with verbal memory performance [48]. A recent study reported cortical shrinkage in the left pars triangularis and pars orbitalis in adults who progress from MCI to severe dementia over time [49]. Moreover, adults with higher levels of education, professional success, and cognitive reserve show a larger volume of pars orbitalis [50] Another study reported a significant positive correlation between the area and volume of pars orbitalis [51]. Similarly, we found adults with a higher area of left pars orbitalis were more likely to be Positive-Agers.

The fifth most important feature was the mean intensity of the thalamus (right hemisphere). The thalamus is located in the center of the brain above the brainstem. It is a midline structure that provides initial processing and is a transitory hub that is connected to the brain as a whole. The thalamus integrates signals from subcortical areas, initially processes them depending on the stimuli involved, and then sends the information to areas of the neocortex for further processing and interpretation [52]. Previous studies observed that amyloid plaques decrease the tissue quality in the thalamus [53]. Altogether, adults with AD and all-cause dementia show lower mean intensity in the thalamus [20, 53]. Similarly, we found that less intensity of the thalamus is associated with a smaller probability of being a Positive-Ager.

Finally, the sixth important feature was the area of the superior frontal gyrus (left hemisphere). People with AD show a significant reduction in cortical surface area in the left hemisphere [54]. Our results support this by showing that adults with less superior frontal area are more likely to be Cognitive Decliners.

The proposed multi-stage feature selection algorithm along with the RF classifier identified that age, education, area of the total surface, inferior frontal, superior frontal, superior temporal gyrus, supramarginal, and volume of presubiculum, LGN, Midbrain as the most influential predictors in distinguishing between Positive-Agers and Cognitive Decliners. The classification pipeline used GC labels as the target variable which incorporates multiple cognitive domains such as processing speed, verbal and numerical reasoning, prospective memory, and visual declarative memory for over 7–9 years. Prediction of cognitive trajectory type in healthy adults using structural brain MRI would be beneficial to find the most resilient and important brain regions in successful cognitive aging and to use this knowledge to identify those at risk of future AD and dementia.

This study has several strengths: first, this study used multiple cognitive domains to create the reflection of GC, while previous studies focused on individual cognitive functions. Second, this study incorporated various sMRI features and allowed the most influential predictors to be identified systematically. Third, we developed a novel feature selection algorithm to find reliable sMRI predictors of cognitive trajectories. The limitations of this study can be listed as the following: first, there was no imaging exam at the baseline visit in UK Biobank, and we could not evaluate the changes in the structural brain MRI from mid- to late-life among the Positive-Agers. Second, the literature identifies multiple significant biomarkers as risk factors for AD, with Apolipoprotein E (APOE) being the major genetic factor among adults over 65. However, the main focus of this study was to identify brain regions that could help distinguish between Positive-Agers and Cognitive Decliners among middle-aged adults rather than improving the classification accuracy. It should be noted that the role of APOE in cognition status among middle-aged adults is not determined. We suggest future studies should examine established risk factors for AD, particularly APOE, as the predictive features of cognitive classes while they consider age range and health conditions. Third, we could not consider medical comorbidities in our analysis due to the following reasons. Participants in UK Biobank tend to be healthier, more financially stable, and better educated than in the UK, USA, and other countries. Therefore, the occurrence of AD, all-cause dementia, and related conditions is very low in the UK Biobank, both due to the relative youthfulness of our sub-cohort but perhaps those factors mentioned. In summary, future studies should investigate medical comorbidities or comorbidity severity to reveal the underlying biomarkers of cognitive decline versus cognitive improvement. Due to the complexity of this problem, we suggest future studies to consider the following: (1) selecting related comorbidities before data collection starts; (2) finding valid criteria for improvement/decline for each comorbidity; and (3) clarifying the reason behind the change if it is associated with the differences between the cognitive classes, or is related to the time interval between baseline and subsequent visits.

## Supplementary Information

Below is the link to the electronic supplementary material.

ESM 1  Supplementary Fig. 1. Feature importance ranking for the final 54 features obtained from the RF classifier and the proposed feature selection algorithm. (PNG 269 KB)

High Resolution Image (TIF 475 KB)

Supplementary file2 (PDF 210 KB)

Supplementary file3 (PDF 300 KB)

## Data Availability

The data used in this study were obtained from the UK Biobank under approved application with Application Number 25057. These data are not publicly available due to restrictions, but they can be accessed by researchers upon application to the UK Biobank (www.ukbiobank.ac.uk) and approval of a research proposal that aligns with the UK Biobank's ethics and access policies.
