# Supplementary figures and images for: A multi-stage feature selection method to improve classification of potential super-agers and cognitive decliners using structural brain MRI data—a UK biobank study
Source: GeroScience. 2024 Dec 10;47(3):3807–19. doi: 10.1007/s11357-024-01458-9 (PMC12181481; doi:10.1007/s11357-024-01458-9)

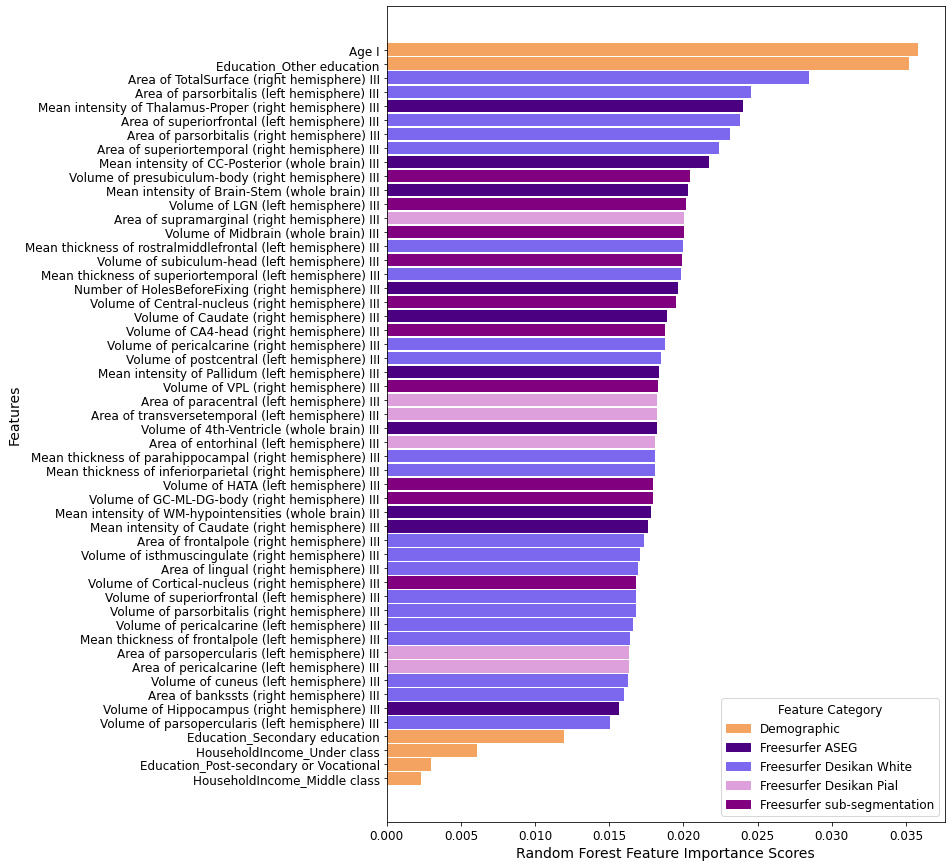

Supplement: Supplementary file 1 — Supplementary Fig. 1. Feature importance ranking for the final 54 features obtained from the RF classifier and the proposed feature selection algorithm. (PNG 269 KB) [file 11357_2024_1458_Fig4_ESM.png]

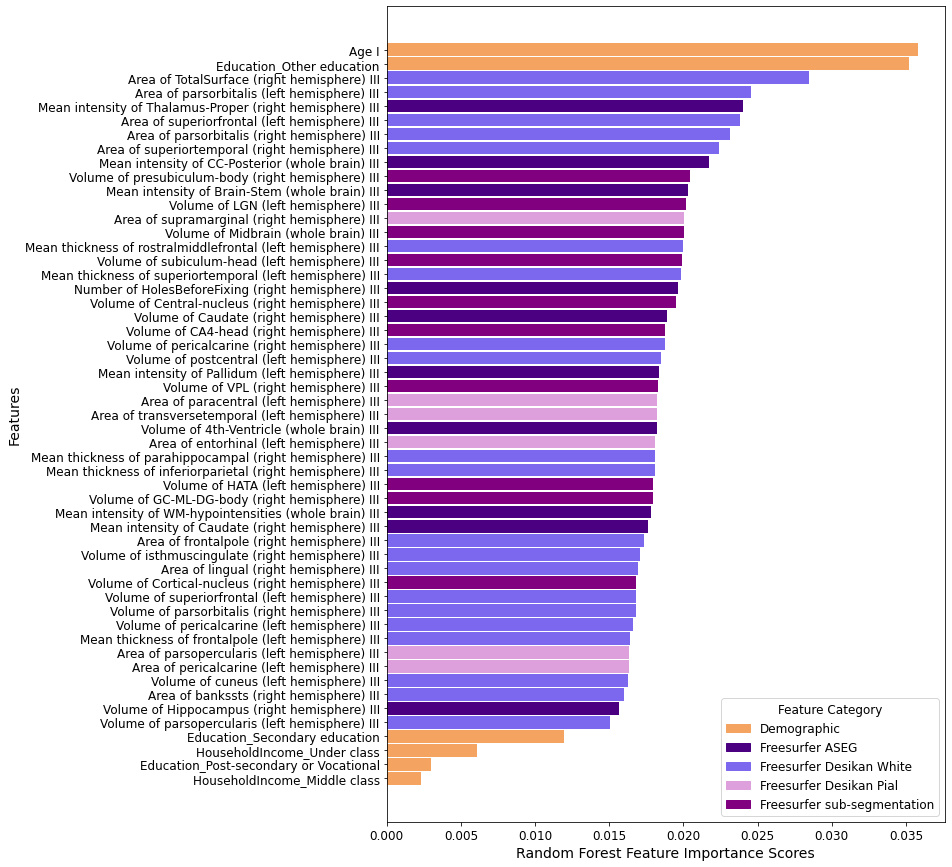

Supplement: Supplementary file 2 — High Resolution Image (TIF 475 KB) [file 11357_2024_1458_MOESM1_ESM.tiff]
