# Supplementary material for: A multi-stage feature selection method to improve classification of potential super-agers and cognitive decliners using structural brain MRI data—a UK biobank study
Source: GeroScience. 2024 Dec 10;47(3):3807–19. doi: 10.1007/s11357-024-01458-9 (PMC12181481; doi:10.1007/s11357-024-01458-9)
Supplement: Supplementary file 3 — Supplementary file2 (PDF 210 KB) [file 11357_2024_1458_MOESM2_ESM.pdf]

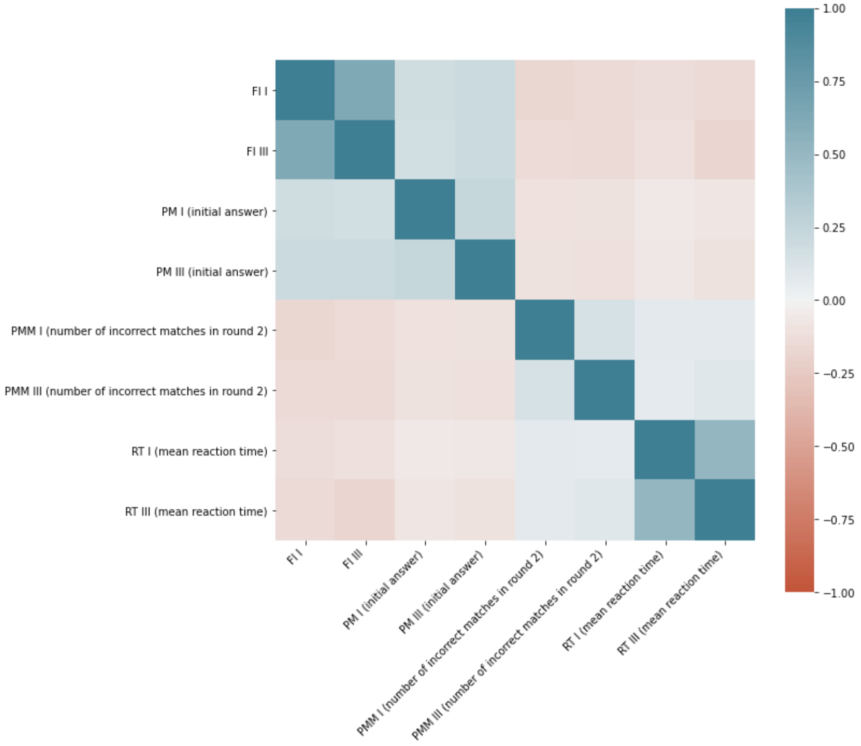

Supplementary Fig. A. 1. Correlation plot for the longitudinal cognitive tests

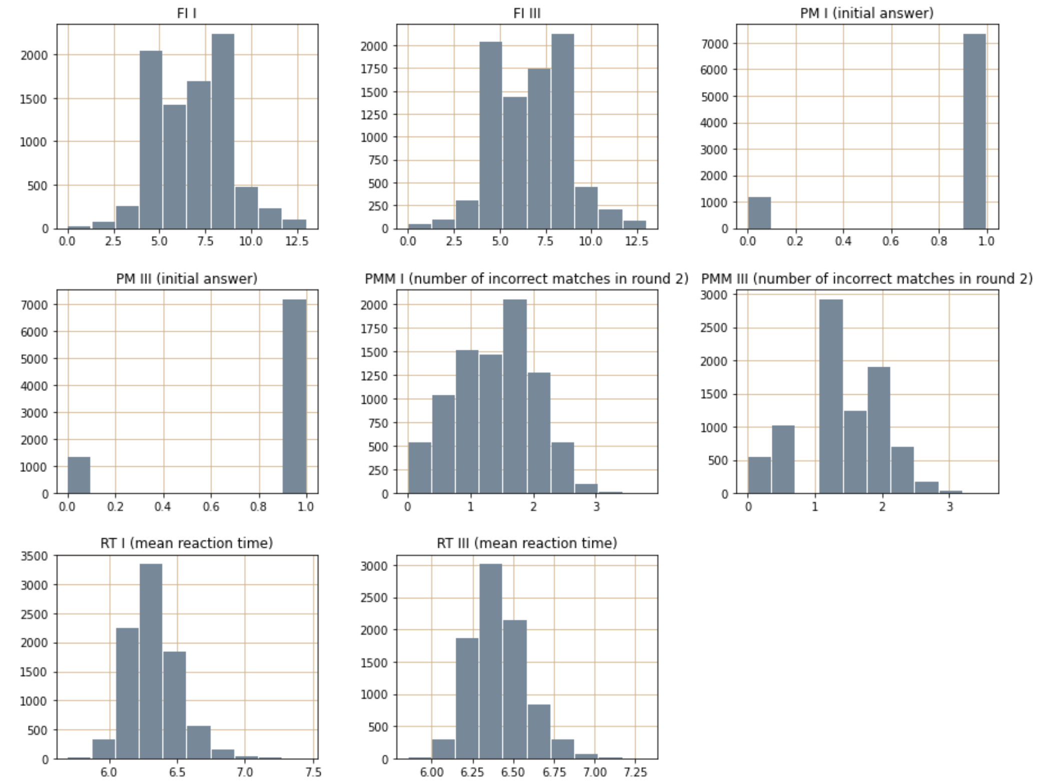

Supplementary Fig. A. 2. Histogram of distributions for the longitudinal cognitive tests

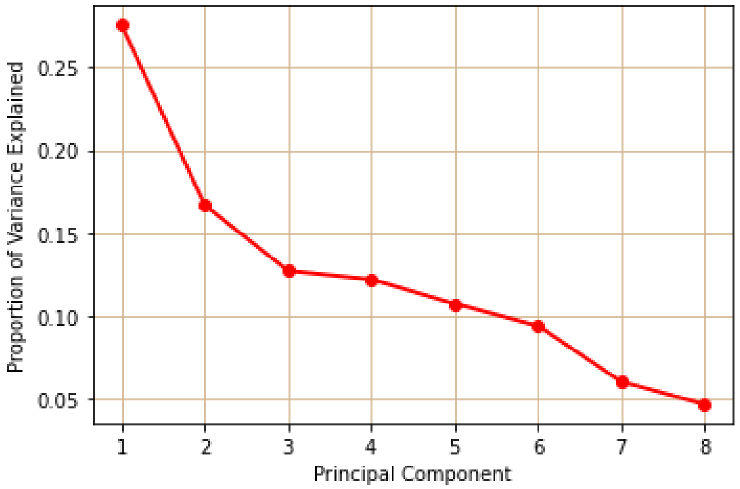

Supplementary Fig. A. 3. Proportion of variance explained by each principal component when applied to cognitive exams.

Supplementary Table A. 1. Loading scores from a principal component analysis on longitudinal cognitive tests (total observations = 6,822 in the training set). Since the sign of weights for FI and PM are negative, and the sign of weights

for PMM and RT are positive, the direction of the GC score is opposite of cognitive performance; that is higher GC scores are associated with lower cognitive performance. The reason is that FI and PMM are positively correlated with cognitive performance and PMM and RT are negatively correlated. To account for this and make the GC score easier to interpret, we multiplied the GC score by -1. This now means that the higher the GC score, the more likely that an individual has better cognitive performance.

**Cognitive Test**	**PC1 [a]**
FI I	-0.49
FI III	-0.49
PM I (initial answer)	-0.29
PM III (initial answer)	-0.31
PMM I (number of incorrect matches in round 2)	0.24
PMM III (number of incorrect matches in round 2)	0.26
RT I (mean reaction time)	0.30
RT III (mean reaction time)	0.35
**% of total variance explained**	**28%**

[a] First principal component of the PCA.

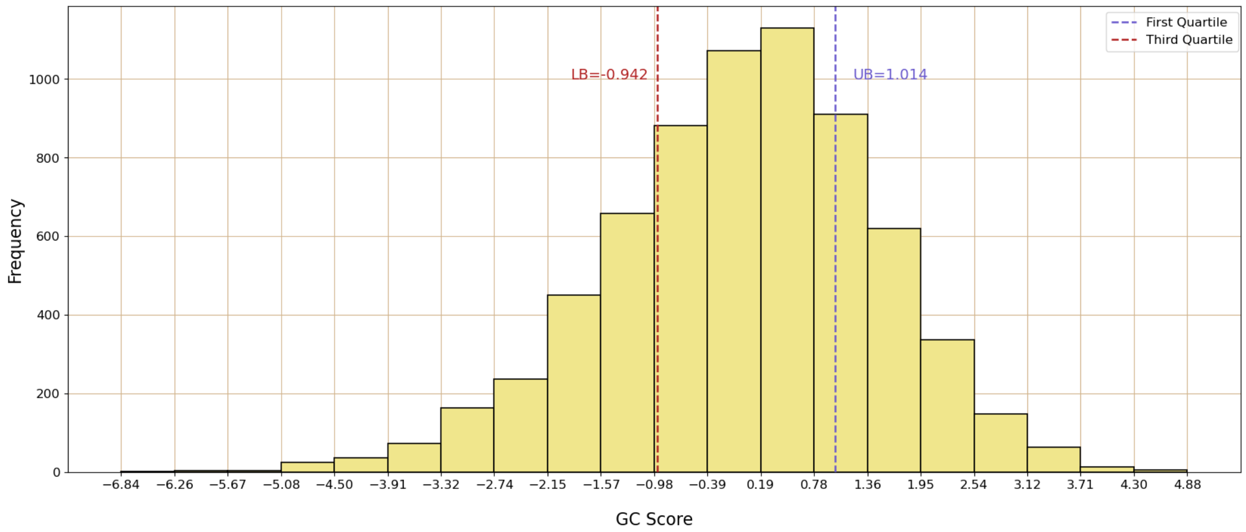

Supplementary Fig. A. 4. Histogram of the composite scores of the first principal component (PC1) (i.e., GC score). Dashed lines show the first and third quartiles that were used as the thresholds to label the participants either Positive-Ager or Cognitive Decliner. Individuals with a composite score of 1.014 and higher are Positive-Agers, and those with composite score of -0.942 and smaller are Cognitive-Decliners.
