# Supplementary material for: A multi-stage feature selection method to improve classification of potential super-agers and cognitive decliners using structural brain MRI data—a UK biobank study
Source: GeroScience. 2024 Dec 10;47(3):3807–19. doi: 10.1007/s11357-024-01458-9 (PMC12181481; doi:10.1007/s11357-024-01458-9)
Supplement: Supplementary file 4 — Supplementary file3 (PDF 300 KB) [file 11357_2024_1458_MOESM3_ESM.pdf]

Supplementary Text B

Multi-Stage Feature Selection Algorithm

Due to the high dimensionality of the datasets, along with other issues covered in the main text, we created a mechanism to select the best subset of features to improve overall model performance. For this purpose, we designed a multi-stage feature selection algorithm that refines the features in three stages and finds the best subset of variables for classification. **Supplementary Fig. B. 1** shows the flowchart of the proposed algorithm.

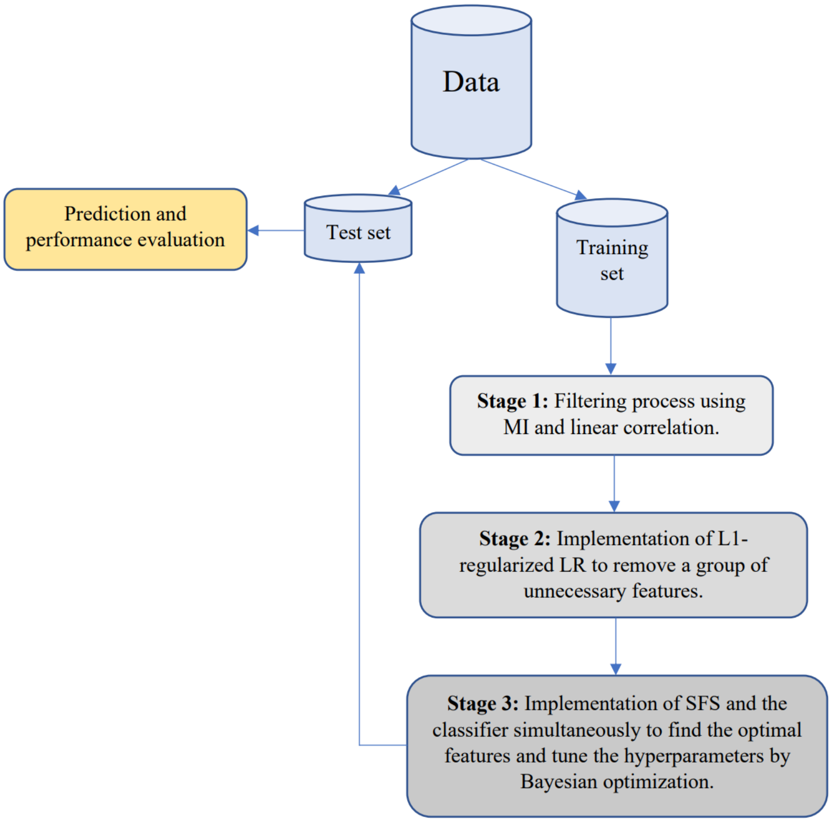

Supplementary Fig. B. 1. Flowchart of the multi-stage feature selection method

Stage A: Let $\{(X_i, y_i): X_i \in R^p, y_i \in R, i = 1, \dots, n\}$ be the training set that has n pairs of a p-length predictor vector and the corresponding value of the response variable. Mutual Information (MI) is a perfect statistic measuring any arbitrary dependency between the random variables. MI determines the amount of information between x and y which is provided about y by observing x or conversely about x by observing y. The MI between two random variables x and y is defined as the following:

$$I(X;\, Y) = H(X) - H(X|Y) = H(Y) - H(Y|X) = H(X) + H(Y) - H(X;Y) \qquad \text{(B. 1)}$$

Where $H(.)$ is the entropy, $H(X|Y)$ and $H(Y|X)$ are the conditional entropies, and $H(X;Y)$ is the joint entropy of x and y. Entropy functions are calculated using probability density functions. One solution to estimate the MI is proposed, which estimates entropy based on an average distance to the k-nearest neighbors [1, 2]. In this study, we specified a threshold for the MI values, denoted as α, and set it as 0.000001 to drop the sMRI features with no significant dependency on the cognitive trajectory type. In the next step of this stage, and to address multicollinearity, we defined a threshold for the correlation between the features, denoted as β. β was set at 0.95 in this study. Within each feature pair with a correlation value higher than β, the feature with the smallest MI score was removed.

Stage B: In the second stage, the L1-regularized Logistic Regression (L1-regularized LR) was used as another stage of filtering to remove features that do not show enough contribution towards predicting the class labels in the training phase. Logistic Regression (LR) is one of the most common classification techniques in machine learning [3]. L1-regularization is widely used in LR models dealing with multicollinearity and overfitting. L1-regularization improves the performance of LR by shrinking the coefficients of the less important or redundant features to zero [4, 5]. Suppose that q features have been removed at **Stage A**, therefore the training set has $p - q$ predictors at the beginning of **Stage B**. In our binary classification problem, $y_i \in \{0, 1\}$ shows the class label: 0 for Cognitive Decliner and 1 for Positive-Ager. Unregularized LR models for the probability distribution of the y are as follows:

$$p(y = 1 | X; \theta) = \sigma(\theta^T X) = \frac{1}{1+\exp(-\theta^T X)} \qquad \text{(B. 2)}$$

Where $\theta \in R^{p-q+1}$ are the coefficients for X or parameters of the LR model. The last column of the matrix X is set to 1 (intercept term is included). $\sigma(.)$ is the sigmoid function. Now, consider the problem of finding the maximum likelihood estimate (MLE) of the parameters θ for the unregularized LR. Therefore, the optimization problem is as follows:

$$\min_{\theta} \sum_{i=1}^{n} -\log p(y_i | X_i;\ \theta) \qquad \text{(B. 3)}$$

Now, let the C regularization parameter for the L1 norm be $C > 0$. The maximum a posteriori (MAP) estimates of θ can be written as follows:

$$\min_{\theta} \sum_{i}^{n} -\log p(y_i | X_i;\ \theta) + C\|\theta\|_1 \qquad \text{(B. 4)}$$

As the value of C decreases, which is the inverse of regularization strength, fewer features get non-zero coefficients. In this study, the L1 regularization is applied to remove redundant or non-significant features. For this purpose, we explored how the change in C affects the mean AUC score. We plotted the cross-validated AUC against the C as it is shown in **Supplementary Fig. B. 2**. As the C gets smaller (up to a certain value $C^* = 0.8$ which is shown by a purple dashed line), the cross-validated AUC score does not improve significantly. Then, when moving from C^* towards zero, the mean AUC score starts to increase with a steep slope. We can observe that the best performance (i.e., highest mean AUC) would be achieved if the regularization value is selected to be $C' = 0.05$, which is shown by a red dashed line. Instead, we let the model's performance decrease in favor of keeping more candidate features. In fact, there is a trade-off between the model's performance and the number of remaining features in the range of $[C', C^*]$, where C^* value is the least strict regularization value that any further reduction does not impact the model's performance afterwards.

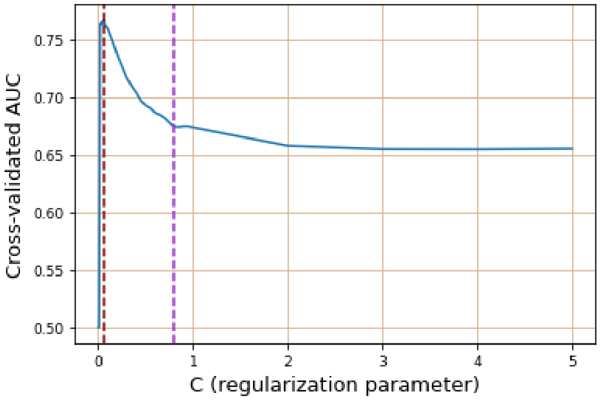

Supplementary Fig. B. 2. Cross validated AUC against C in L1-regularized LR. Red and purple dashed lines correspond to the C' and C^*, respectively.

Supplementary Fig. B. 3 shows the effect of different values of C on various metrics including AUC, accuracy, precision, and recall on the sMRI features. As shown with the purple dashed line, selecting the value of C and any of

the other metrics than AUC results in a similar interpretation. All these metrics support that $C^* = 0.8$ is a good candidate for this dataset.

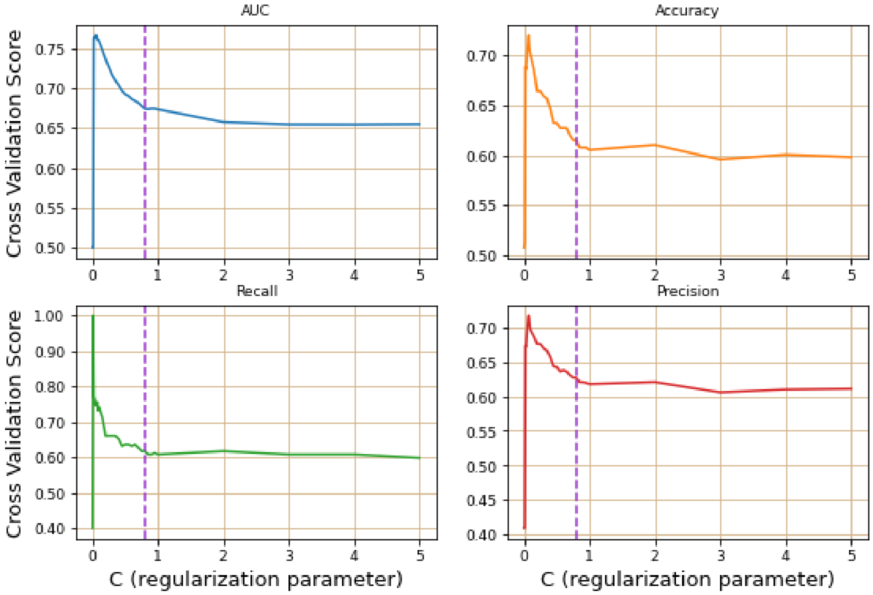

Supplementary Fig. B. 3. Cross-validated AUC, accuracy, recall, and precision against the C in L1-regularized LR

Stage C: In the last stage of the algorithm, Sequential Feature Selection (SFS) is employed using a forward selection approach. SFS starts with an empty set of features and incrementally adds the best features in each iteration [6]. We used SFS as the final feature selector for our classification problem. The proposed algorithm utilizes Bayesian Optimization (BO) to tune the parameters in our algorithm. BO is a common method to optimize objective functions with unknown structures and high computational costs. This technique is widely used to tune hyper-parameters in machine learning models. In this study, we maximized AUC in the objective function to tune the hyper-parameters. The parameters optimized with BO in this study are the number of selected features in SFS and the hyperparameters of the final classifier through 5-fold cross-validation.

1. Effect of regularization parameter on the model's performance

By setting the C as 0.8, the number of features drops from 354 to 286. Thus, the dimension of input features to Stage III of the algorithm decreases significantly, leading to better performance and faster runtime. If we pick a C smaller than 0.8, more features are removed, and the final classification performance will be worse. On the other hand, if we pick a C bigger than 0.8, fewer features are removed, and the final classification performance will be comparable or worse. To show the optimality of this decision, we examined different values of C and reported the results in the **Supplementary Table B. 1**.

Supplementary Table B. 1. Evaluation of the proposed algorithm for different values of C on sMRI features

C	N_1	N_2	N_3	Time (s)	Accuracy	Precision	Recall	AUC
0.05*	354	126	66	18592.63	66	67	75	70
0.4	354	251	48	38726.22	66	68	75	71
0.8	354	286	54	47591.11	**67**	**68**	**79**	**73**
2.5	354	331	52	53819.45	65	67	74	71
5.0	354	345	64	64783.56	65	66	73	70

N_1 represents the number of features by the end of Stage A
N_2 represents the number of features by the end of Stage B
N_3 represents the number of features by the end of Stage C
* This value resulted in the highest AUC in Stage B.

In summary, L1-regularized LR is a time-efficient model for feature selection, but it selected too many features. This led to mediocre classification performance. On the other hand, SFS selected a small group of features leading to good prediction performance, but it was not time efficient. Using the L1-regularized LR with the proposed procedure dropped unnecessary features and gave the smaller input features to the SFS to find the optimal feature subset in a significantly shorter time.

References

[1] Ross BC. Mutual Information between Discrete and Continuous Data Sets. PLoS One 2014;9:e87357. https://doi.org/10.1371/JOURNAL.PONE.0087357.

[2] Amiri F, Rezaei Yousefi M, Lucas C, Shakery A, Yazdani N. Mutual information-based feature selection for intrusion detection systems. Journal of Network and Computer Applications 2011;34:1184–99. https://doi.org/10.1016/J.JNCA.2011.01.002.

[3] Yang S, Shapiro LG, Cunningham ML, Speltz M, Lee S-I. Classification and Feature Selection for Craniosynostosis 2011.

[4] Musa AB. A comparison of ℓ1-regularizion, PCA, KPCA and ICA for dimensionality reduction in logistic regression. International Journal of Machine Learning and Cybernetics 2014;5:861–73. https://doi.org/10.1007/S13042-013-0171-7/METRICS.

[5] El Guide M, Jbilou K, Koukouvinos C, Lappa A. Comparative study of ¿η regularized logistic regression methods for variable selection. Commun Stat Simul Comput 2020;2020:1–16. https://doi.org/10.1080/03610918.2020.1752379/SUPPL_FILE/LSSP_A_1752379_SM6951.PDF.

[6] Somol P, Novovicová J, Pudil P, CZ37701 JH. Improving sequential feature selection methods performance by means of hybridization. InProc. 6th IASTED Int. Conf. on Advances in Computer Science and Engrg. ACTA Press 2010 Mar (Vol. 2010). https://doi.org/10.2316/P.2010.689-001.
